# Feeding a whole-cell inactivated *Pichia guilliermondii* yeast to gestating and lactating sows in a commercial production system

**DOI:** 10.1093/tas/txac160

**Published:** 2022-12-06

**Authors:** Morgan T Thayer, Ricardo M Garcia, Alan W Duttlinger, Julie A Mahoney, Allan P Schinckel, Matthew D Asmus, Daniel B Jones, Jim L Dunn, Brian T Richert

**Affiliations:** Department of Animal Sciences, College of Agriculture, Purdue University, West Lafayette, IN, USA; Department of Animal Sciences, College of Agriculture, Purdue University, West Lafayette, IN, USA; Department of Animal Sciences, College of Agriculture, Purdue University, West Lafayette, IN, USA; Department of Animal Sciences, College of Agriculture, Purdue University, West Lafayette, IN, USA; Department of Animal Sciences, College of Agriculture, Purdue University, West Lafayette, IN, USA; ADM Animal Nutrition, Quincy, IL, USA; ADM Animal Nutrition, Quincy, IL, USA; ADM Animal Nutrition, Quincy, IL, USA; Department of Animal Sciences, College of Agriculture, Purdue University, West Lafayette, IN, USA

**Keywords:** born alive, sows, yeast

## Abstract

A total of 606 sows (PIC 1050) and their progeny (PIC 1050 × 280) were used to determine if feeding gestating and lactating sows a proprietary strain of *Pichia guilliermondii* as a whole-cell inactivated yeast product (**WCY**; CitriStim, ADM Animal Nutrition, Quincy, IL) improves sow and litter performance in a commercial production system. Once confirmed pregnant at d 35 post-breeding pregnancy check, sows were fed a basal gestation control (**CON**) diet (0.55% SID lysine) or the control diet fortified with 0.15% of the WCY replacing corn in the CON diet. Dietary treatments were also fed in lactation (1.05% SID lysine) once sows were moved into farrowing crates on approximately d 112 of gestation until weaning. Sows supplemented with WCY in gestation and lactation had increased total born piglets by 0.45 pigs (*P* < 0.04), piglets born alive (14.27 vs. 13.85; *P* < 0.04), and, therefore, heavier born alive litter weights (*P* < 0.001) compared to CON fed sows. A greater post cross-foster litter size (*P* < 0.001) meant that litter size at weaning was increased by 0.54 pigs when sows were fed WCY compared to CON (*P* < 0.001). However, litter weaning weights and 21-d adjusted litter weaning weights were similar (*P* > 0.158), although numerically greater, for WCY fed sows. Pigs from CON fed sows were 0.35 kg heavier at weaning compared to pigs from WCY fed sows (*P* < 0.001). This increase in weaning weight of pigs from CON fed sows is partially explained by their 0.93 d longer lactation (*P* < 0.001) and may also be due to the smaller litter size throughout lactation. The percent of litters treated for scours decreased from 38.3 to 14.2% when sows were fed WCY (*P* < 0.001). The distribution of birth and weaning weights was not impacted (*P* > 0.2461) by treatment. In conclusion, feeding gestating and lactating sows a proprietary strain of *Pichia guilliermondii* as a whole-cell inactivated yeast product increased the number of pigs born and weaned, and decreased the prevalence of scours during lactation.

## INTRODUCTION

Dietary yeast products fed to swine have been identified as potential growth promoting feed additives and have been reported to have beneficial effects on the pig’s immune system and health (Shen et al., 2011). *Saccharomyces cerevisiae* is a common yeast species that has been studied. However, *Saccharomyces cerevisiae* has a larger cell surface area and weaker hydrophobic properties compared to *Pichia guilliermondii* and physical differences may provide *Pichia guilliermondii* a stronger ability to inhibit pathogenic bacteria from binding to small intestinal epithelium of swine and broiler chickens compared to *Saccharomyces cerevisiae* (Peisker et al., 2017). A proprietary strain of *Pichia guilliermondii* as a whole-cell inactivated yeast (WCY) is a co-product of citric acid fermentation and was fed in this experiment.

The yeast cell wall fraction of many yeast species represents approximately 20–30% of the dry weight of the yeast cell and consists of 85–90% polysaccharide and 10–15% crude protein (Lesage and Bussy, 2006; Nguyen et al., 1998). Specifically, yeast cell walls are a source of mannan-oligosaccharides and β-glucans. Mannan-oligosaccharides positively impact gut health by beneficially shifting bacterial populations in the gut by binding bacterial pathogens in the intestine and preventing their adhesion to host epithelium (Kogan and Kocher, 2007). Beta-glucans bind membrane receptors of innate immune cells, located in the small intestinal mucosal lining, which signals the immune system to prepare for potential pathogenic threats (Baert et al., 2015; Batbayar et al., 2012). Therefore, we hypothesized that the overall reduction in pathogenic stressors to the sow, the potential shift in gut microbiota, and immune system activation would improve sow reproductive performance and her progeny’s pre-weaning performance.

The objective of this study was to determine if feeding gestating and lactating sows a proprietary strain of *Pichia guilliermondii* as a whole-cell inactivated yeast product (CitriStim, ADM Animal Nutrition, Quincy, IL) would improve sow and litter performance in a commercial production system. Performance parameters included litter characteristics at birth, birth weights individually and/or whole litter weights, individual and/or whole litter weaning weights, survival rate, and percent of litters treated for scours.

## MATERIALS AND METHODS

### General

The experimental procedures used in this study were approved by the Purdue Animal Care and Use Committee (PACUC # 1909001949). This study was conducted at Martin Family Farms (Williamsport, IN) breed-to-wean commercial facility from May to September of 2018. General farm staff were blinded to dietary treatments throughout the study. Feed samples were analyzed by ADM Animal Nutrition (Quincy, IL).

### Animals and Diets

A total of 606 sows (PIC 1050) and their progeny (PIC 1050 × 280) were used in this experiment. Average sow parity was 3.55 for this experiment and ranged from gilts to parity 7. Sows were bred and maintained in stalls until ultrasound confirmation of pregnancy on d 35 post-breeding. Pregnant sows were then moved to group pens and the whole pen of sows was assigned to one of two dietary treatments:

1) **CON**- Control diet
2) **WCY**- CON diet + 0.15% whole-cell inactivated yeast product

The WCY (CitriStim, ADM Animal Nutrition, Quincy, IL) was added at the expense of corn. Treatments were fed from d 35 of gestation through lactation.

Gestation group pens used electronic sow feeders (AP Schauer ESF Stations, AGCO, Duluth, GA) that delivered approximately 2.27 kg of feed per sow per day, varying based on the individual sow’s body condition. Industry standard diet formulations were targeted to meet or exceed swine nutrient requirements (NRC, 2012) for gestating and lactating sows (Table 1). The monthly average high temperatures when these sows were gestating were 27.3, 27.6, 28.0, 27.7, and 26.7°C from May to September of 2018, respectively.

**Table 1.** Sow diet composition (as-fed basis)^1^.

Ingredient, %	Control (CON)		CitriStim (WCY)	
	Gestation^2^	Lactation^3^	Gestation^2^	Lactation^3^
Corn	67.03	56.93	66.88	56.78
Soybean meal, 47% CP	4.56	26.89	4.56	26.89
DDGS, 7% Fat	22.50	10.00	22.50	10.00
Choice white grease	------	2.50	------	2.50
Soybean Hulls	2.50	------	2.50	------
Limestone	1.52	1.35	1.52	1.35
Monocalcium Phos. (21% P)	0.77	0.86	0.77	0.86
Salt	0.05	0.11	0.05	0.11
L-Lysine-HCl	0.28	0.28	0.28	0.28
L-Threonine	0.03	0.07	0.03	0.07
L-Tryptophan	0.01	------	0.01	------
Vitamin and Trace Mineral Premix^4^	0.60	0.60	0.60	0.60
Sow Mineral Pack^5^	------	0.25	------	0.25
Choline Chloride (60%)	0.10	0.10	0.10	0.10
Visano Sow^6^	0.05	0.05	0.05	0.05
NAT-P-E 2500^7^	------	0.01	------	0.01
CitriStim^8^	------	------	0.15	0.15
Total	100.00	100.00	100.00	100.00
*Calculated analysis* ^9^				
ME, Kcal/kg	3076.8	3238.7	3076.8	3238.7
Crude Protein, %	13.25	19.31	13.25	19.31
Crude Fat, %	3.64	5.65	3.64	5.65
Digestible lysine, %	0.55	1.05	0.55	1.05
Digestible lysine:ME, g/Mcal	1.79	3.24	1.79	3.24
Digestible met+cys:lysine, %	70.00	50.00	70.00	50.00
Digestible thr:lys, %	70.00	63.00	70.00	63.00
Digestible tryp:lys, %	18.00	18.50	18.00	18.50
Ca, %	0.85	0.85	0.85	0.85
P, %	0.56	0.59	0.56	0.59
Available P, %.	0.45	0.45	0.45	0.45
*Analysis* ^10^				
Crude Protein, %	12.71	20.60	13.63	19.77
Crude Fat, %	3.70	4.70	3.44	5.10
Crude Fiber, %	3.83	3.07	4.05	3.36
Ash, %	4.50	5.16	4.83	4.98
Moisture, %	13.08	14.68	12.49	12.29

^1^Formulations were completed by United Animal Health (Sheridan, IN).

^2^Diets were fed from d 35 post-breeding until they were loaded into farrowing crates on approximately d 112 of gestation.

^3^Diets were fed from d 112 of gestation until weaning.

^4^Provided per kg of diet: vitamin A, 11,161 IU; vitamin D_3_, 2,545 IU; vitamin E, 66 IU; vitamin K, 1.42 mg; riboflavin, 6.6 mg; pantothenic acid, 23.6 mg; niacin, 44.2 mg; B_12_, 31µg; biotin, 0.44 mg; folic acid, 1.62 mg; thiamine, 0.25 mg; pyrdoxine-B_6_, 0.25 mg; iron, 129 mg; zinc, 125 mg; manganese, 60 mg; copper, 20 mg; iodine, 1.26 mg; selenium, 0.3 mg; cobalt, 0.02 mg; calcium, 0.095%; sodium, 0.08%, chloride, 0.12%; phytase, 371 FTU; and estimated 0.12% phosphorus release from the phytase.

^5^Provided per kg of diet: potassium, 0.057%; magnesium, 0.012%; sodium, 0.012%; sulfur, 0.030%; chloride, 0.039%.

^6^Visano Sow is a direct fed microbial (DFM) for sows from United Animal Health. It is a multi-strain *Bacillus spp.*

^7^Natuphos E phytase enzyme (*Aspergillus niger*; 2500 FTU/g, BASF, Florham Park, NJ) provided 250.7 FTU/kg phytase activity in lactation diets.

^8^CitriStim (ADM Animal Nutrition, Quincy, IL) is a proprietary strain of *Pichia guilliermondii*, a whole-cell inactivated yeast product. Nutrient value was assumed to be equal to corn that it replaced in the control diet.

^9^Calculated nutrients were targeted to meet or exceed the NRC 2012. Nutrient Requirements of Swine. 11th ed. Natl. Acad. Press, Washington, DC.

^10^ Samples were collected at Martin Family Farms (Williamsport, IN), subsampled at Purdue University, and shipped to ADM Animal Nutrition (Quincy, IL) for analysis.

Sows were moved into individual farrowing crates on approximately d 112 of gestation and allowed ad libitum access to lactation feed. Each farrowing crate was equipped with a dry feeder that was fed by a continuous feed line. Sows and litters used in this experiment had a minimum of 8 pigs born alive and sows were parity 7 or lower. Parity was equalized across treatments. Sows were selected on a rolling basis over the duration of 4 weeks so that the parity distribution of the experiment resembled the farm’s parity distribution from their electronic records program (PigKnows; Greeley, CO). The number of parity 2 sows for data collection were limited near the end of the data collection period. Therefore, additional parity 1, 3, and 4 sows were selected to replace the unavailable parity 2 sows (Table 4).

**Table 4.** Effects of parity when feeding a whole-cell inactivated yeast product (**WCY**) to gestating and lactating sows on sow and whole litter performance in a commercial production system^1,2^ (606 sows; corresponding to Table 2 results).

	Parity							SEM	Probability, *P* <
	1	2	3	4	5	6	7		
Sows, *n*	135	81	96	86	81	72	55	---	---
Percent of sows^3^	22.3	13.4	15.8	14.2	13.4	11.9	9.1	---	---
Litter characteristics									
Total born,^4^*n*	13.70^d^	14.98^abc^	15.64^ab^	14.97^abc^	15.75^a^	14.74^bc^	15.23^abc^	0.363	< 0.001
Born alive, *n*	13.15^e^	14.28^abc^	14.77^a^	14.06^abcd^	14.76^ab^	13.54^cde^	13.83^cde^	0.336	< 0.001
Stillborn,^5^*n*	0.55^c^	0.70^bc^	0.87^ab^	0.91^ab^	0.99^ab^	1.21^ab^	1.40^a^	0.175	0.001
Stillborn,^5^ %	3.65^d^	4.30^cd^	5.22^bc^	5.59^abc^	6.21^abc^	7.36^abc^	8.88^a^	1.024	0.002
Mummies,^5^ n	0.21	0.28	0.38	0.42	0.28	0.49	0.25	0.102	0.244
Born alive litter weight, kg	17.33^f^	20.90^ab^	20.98^a^	20.27^abc^	19.38^cde^	18.21^f^	18.32^def^	0.441	< 0.001
12-hour measurements^6^									
Litter size, *n*	12.80^d^	13.81^abc^	14.16^a^	13.38^bcd^	14.06^ab^	12.73^d^	13.41^abcd^	0.326	< 0.001
Survival before cross-foster,%	97.49^a^	96.85^abc^	95.96^abcd^	95.49^bcd^	95.41^bcd^	94.70^d^	96.99^ab^	0.889	0.049
Litter weight, kg	17.20^e^	20.64^a^	20.61^ab^	19.83^abc^	19.08^cd^	17.75^e^	18.10^de^	0.448	< 0.001
Post cross-foster litter size, *n*	13.57^abc^	13.85^ab^	14.08^a^	13.20^c^	13.58^abc^	12.99^c^	13.12^c^	0.274	0.005
Weaning measurements^7^									
Sows weaned, *n*	131	80	95	85	79	67	52	---	---
Weaning age	18.76	19.21	18.87	18.96	19.06	18.96	18.87	0.096	0.092
Litter size, *n*	12.13^ab^	12.54^a^	12.06^abc^	11.67^bcde^	11.84^bcd^	11.33^def^	10.96^f^	0.234	< 0.001
Survival after cross-foster, %	89.72^ab^	90.85^a^	86.36^c^	88.98^abc^	87.56^abc^	87.39^abc^	85.25^c^	1.546	0.029
Litter weight, kg	57.63^f^	71.27^a^	68.02^abc^	69.02^ab^	65.68^bcd^	63.41^de^	59.76^ef^	1.549	< 0.001
21-d adjusted litter weight,^8^ kg	64.52^e^	78.42^a^	75.82^ab^	76.71^a^	72.68^bc^	70.45^cd^	66.66^de^	1.685	< 0.001
Piglet body weight,^9^ kg									
Born alive	1.35^d^	1.50^a^	1.46^abc^	1.49^ab^	1.35^d^	1.40^cd^	1.35^d^	0.028	< 0.001
Weaning^7^	4.79^c^	5.72^ab^	5.69^b^	5.95^a^	5.55^b^	5.63^b^	5.46^b^	0.106	< 0.001
21-day adjusted weaning	5.35^c^	6.29^b^	6.34^b^	6.60^a^	6.14^b^	6.25^b^	6.08^b^	0.114	< 0.001
Litter treated for scours,^10^ %	59.73^a^	27.31^b^	23.67^bc^	19.49^bc^	22.37^bc^	12.84^c^	18.40^bc^	5.521	< 0.001

^a,b,c,d,e,f^Means within a row without common superscripts differ (*P* < 0.05).

^1^ A total of 606 sows and their progeny (PIC 1050 × 280) were used to determine if feeding a whole-cell inactivated yeast product (WCY; ADM Alliance Nutrition, Inc., Quincy, IL), to sows in gestation and lactation diets influences sow and pre-weaning pig performance in a commercial production system.

^2^ Two maternal dietary treatments were fed. In gestation, post pregnancy check on d 35, and in lactation, sows were fed a control basal diet or the basal diet with 0.15% added WCY replacing corn.

^3^ The farm’s parity distribution over the previous 6 months before the study started was: 20% parity 1, 18% parity 2, 12% parity 3, 12% parity 4, 11% parity 5, 11 % parity 6, 8% parity 7, and 8% parities 8 to 11 (PigKnows; Greeley, CO).

^4^ Total born was calculated as born alive plus stillborn piglets and does not include mummies.

^5^ Data were square root-transformed to meet assumptions of normality; however, means and standard errors are presented as non-transformed values for ease of interpretation and p-values represent the square root-transformed data.

^6^ The 12-hour measurements were collected between 1 and 12 hours of birth from living piglets and before cross-fostering. The 12-hour survival rate does not include stillborn mortality.

^7^ Weaning weights were collected 1 day before actual weaning age.

^8^ Due to a significant difference in weaning age, weaning litter weights were adjusted for weaning age to a 21-day basis using adjustment factors from the Guidelines for Uniform Swine Improvement Programs 10.2.1 written by the National Swine Improvement Federation in December 1987.

^9^ Piglet body weights were calculated by dividing the litter weight by the number of pigs in the litter. Born alive is the litter weight at birth not including stillborn pigs. The 12-hour weight is the litter weight before cross-fostering, which would exclude any crushed or non-viable pigs at that time. Weaning is the litter weight at weaning. The 21-day adjusted weaning is the 21-day adjusted litter weight divided by the number of pigs at weaning.

^10^ Percent of litters treated for scours at least 1 time.

Whole litter weights were collected on the day of birth (d 0) and on the day before weaning from approximately 200 litters per treatment. An additional 100 litters per treatment were tagged and weighed on an individual piglet basis on d 0 and the day before weaning. Sow and litters were housed in 28 different farrowing rooms, with each room being fed a single diet (all from the same gestation pen and diet) to minimize potential feeding errors and keeping gestation contemporary groups together. Individual (model BS-1818-50-NO) and litter (model BS-2424-200-NO) weights were collected using Avery Weigh-Tronix digital scales (Fairmont, MN). Two check weights (5 and 22.7 kg) were used to check the scales for accuracy before the start of each weigh day.

Day 0 care included an oral dose of Marquis (15% w/w ponazuril, Merial, Inc., Duluth, GA), 0.2 mL injectable Excede (ceftiofur crystalline free acid, Zoetis, Parsippany, NJ), and 2.0 mL injectable iron (100 mg/mL). The extremely low birth weight pigs under 500 grams were considered non-viable and euthanized on d 0. Cross-fostering within treatments occurred on d 0 after processing and weights to equalize litter size within treatment. If litters were scouring, SpectoGard (spectinomycin, Bimeda, Dublin, Ireland) was administered orally to individual pigs between days 1 and 3 post-farrowing depending on the severity of diarrhea (2 mL given once daily). Castration and tail docking occurred between d 3 and 7 post-farrowing depending on labor availability. Pigs were weaned 3 days per week and whole rooms with the oldest litters were weaned first. Once nearing the contracted number of pigs each weaning day, the oldest pigs out of the remaining rooms were weaned with no consideration of dietary treatment.

### Chemical Analyses

All diets were formulated by United Animal Health (Sheridan, IN). Gestation and lactation diet samples were collected at the sow farm and subsampled at Purdue University then shipped to ADM Animal Nutrition (Quincy, IL) for analysis. Feed samples were analyzed (Table 1) for crude protein (AOAC 990.03, 2006), crude fat (AOAC 920.39, 2006), crude fiber (AOCS Ba6a-05, 2006), ash (AOAC 942.05, 2006), and moisture content (Shreve et al., 2006).

### Statistical Analyses

Data were analyzed as a completely randomized design using the MIXED procedure in SAS 9.4 (SAS Institute, Inc., Cary, NC) with sow or piglet as the experimental unit. For sow, litter, and individual piglet performance, dietary treatment of the sow and parity (1 to 7) were fixed effects. Stillborn and mummy data were square root-transformed to meet assumptions of normality. When modeling individual birth weights, dietary treatment and parity group (parity 1, 2, and 3+) were included as fixed effects with total born used as a covariate. When modeling adjusted 21-d weaning weights of individual piglets, dietary treatment, parity group, birth weight within treatment, post cross-foster litter size, and if the piglet was cross-fostered or not were all included as fixed effects (National Swine Improvement Federation, 1987). The GLM procedure in SAS 9.4 was used to model adjusted 21-d weaning weights of individual piglets, including birth weight and dietary treatment as independent variables. The FREQ procedure in SAS 9.4 was used to generate a frequency plot and chi-square analysis of the distribution of birth and weaning weights separated by dietary treatment. To assess differences among parities the PDIFF option of the LS Means function was used for multiple comparisons among parities. Differences were considered significant at *P* ≤ 0.05 and trends at 0.05 < *P* ≤ 0.10.

## Results

### Dietary Treatment Effects

There were no dietary treatment × parity interactions for sow, litter, and individual nursing pig performance (*P* > 0.05; Tables 2 and 3). Therefore, the interaction was removed from the model. Results presented in Table 2 includes data from all 606 sows and litters used in this experiment. Approximately 2/3 of the litters were weighed on a whole litter bases, with the remaining 1/3 of the litters being weighed on an individual pig basis. Sows supplemented with WCY in gestation and lactation had more total pigs born (0.45 pigs; *P* < 0.04), more pigs born alive (0.42 pigs; *P* < 0.04), and 1.02 kg heavier litter weights at birth compared to CON fed sows (*P* < 0.001; Table 2). At 12 h after birth, there was no difference (*P* > 0.05) in survival rate between treatments, and, therefore, WCY fed sows had 0.44 more pigs per litter (*P* < 0.027) and litters were 0.99 kg heavier (*P* < 0.001) compared to CON fed sows. Post cross-foster litter size was also greater (*P* < 0.001) for WCY fed sows by 0.68 pigs, and stayed higher by 0.54 pigs at weaning compared to CON fed sows (*P* < 0.001).

**Table 2.** Effects of feeding a whole-cell inactivated yeast product (**WCY**) to gestating and lactating sows on sow and whole litter performance in a commercial production system^1^.

	Diet ^2^		SEM	Probability, *P* <	
	Control (CON)	CitriStim (WCY)		Diet	Parity
Sows, *n*	300	306	---	---	---
Parity	3.53	3.57	---	---	---
Litter characteristics					
Total born,^3^ n	14.78	15.23	0.158	0.040	< 0.001
Born alive, n	13.85	14.27	0.146	0.039	< 0.001
Born alive, %	94.17	94.04	0.447	0.832	< 0.001
Stillborn,^4^*n*	0.93	0.96	0.076	0.492	0.001
Stillborn,^4^ %	5.82	5.96	0.441	0.530	0.002
Mummies,^4^ n	0.33	0.33	0.045	0.458	0.244
Born alive litter weight, kg	18.83	19.85	0.192	< 0.001	< 0.001
12-hour measurements^5^					
Litter size, n	13.26	13.70	0.142	0.027	< 0.001
Survival before cross-foster, %	96.00	96.25	0.387	0.633	0.049
Litter weight, kg	18.53	19.52	0.195	< 0.001	< 0.001
Post cross-foster litter size, n	13.14	13.82	0.120	< 0.001	0.005
Weaning measurements^6^					
Sows weaned, n	295	294	---	---	---
Weaning age	19.42	18.49	0.062	< 0.001	0.092
Litter size, n	11.52	12.06	0.100	< 0.001	< 0.001
Survival after cross-foster, %	88.44	87.59	0.663	0.354	0.029
Litter weight, kg	65.54	64.41	0.664	0.220	< 0.001
21-day adjusted litter weight,^7^ kg	71.47	72.89	0.722	0.158	< 0.001
Piglet body weight,^8^ kg					
Born alive	1.40	1.43	0.012	0.125	< 0.001
12-hour	1.41	1.44	0.012	0.122	< 0.001
Weaning^6^	5.71	5.36	0.045	< 0.001	< 0.001
21-day adjusted weaning	6.24	6.06	0.049	0.012	< 0.001
Litter treated for scours,^9^ %	38.33	14.18	2.406	< 0.001	< 0.001

^1^ Production performance of 606 sows and their progeny (PIC 1050 × 280) was recorded to determine if feeding a whole-cell inactivated yeast product (WCY; ADM Alliance Nutrition, Inc., Quincy, IL), to sows in gestation and lactation diets influences sow and pre-weaning pig performance in a commercial production system. Data were analyzed as a completely randomized design using the MIXED procedure in SAS 9.4 (SAS Institute, Inc., Cary, NC) with sow or piglet as the experimental unit. For sow, litter, and individual piglet performance, dietary treatment of the sow and parity (1 to 7) were fixed effects. No dietary treatment ×parity interaction was observed and therefore, the interaction was removed from the model.

^2^ Two maternal dietary treatments were fed. In gestation, beginning at post pregnancy check on d 35, and in lactation, sows were fed a control basal diet or the basal diet with 0.15% added WCY replacing corn.

^3^ Total born was calculated as born alive plus stillborn piglets and does not include mummies.

^4^ Data were square root-transformed to meet assumptions of normality; however, means and standard errors are presented as non-transformed values for ease of interpretation and p-values represent the square root-transformed data.

^5^ The 12-hour measurements were collected between 1 and 12 hours of birth from living piglets and before cross-fostering. The 12-hour survival rate does not include stillborn mortality.

^6^ Weaning weights were collected 1 day before actual weaning age.

^7^ Due to a significant difference in weaning age, weaning litter weights were adjusted for weaning age to a 21-day basis using adjustment factors from the Guidelines for Uniform Swine Improvement Programs 10.2.1 written by the National Swine Improvement Federation in December 1987.

^8^ Piglet body weights were calculated by dividing the litter weight by the number of pigs in the litter. Born alive is the live litter weight at birth not including stillborn pigs. The 12-hour weight is the litter weight before cross-fostering, which would exclude any crushed or non-viable pigs at that time. Weaning is the litter weight at weaning. The 21-day adjusted weaning is the 21-day adjusted litter weight divided by the number of pigs at weaning.

^9^ Percent of litters treated for scours at least 1 time.

**Table 3.** Effects of feeding a whole-cell inactivated yeast product (**WCY**) to gestating and lactating sows on sow and individual piglet performance in a commercial production system^1^.

	Diet ^2^		SEM	Probability, *P* <	
	Control (CON)	CitriStim (WCY)		Diet	Parity
Sows, *n*	105	106	---	---	---
Parity	3.55	3.58	---	---	---
Litter characteristics					
Total born,^3^*n*	15.00	15.40	0.284	0.320	0.005
Born alive, *n*	14.16	14.39	0.256	0.515	0.012
Born alive, %	94.91	93.95	0.820	0.399	0.526
Stillborn,^4^*n*	0.85	1.01	0.147	0.378	0.207
Stillborn,^4^ %	5.09	6.05	0.820	0.375	0.192
Mummies,^4^ n	0.34	0.30	0.070	0.887	0.936
Born alive litter weight, kg	19.24	20.10	0.332	0.063	< 0.001
12-hour measurements^5^					
Litter size, *n*	13.55	13.86	0.248	0.378	0.044
Survival before cross-foster, %	96.01	96.49	0.615	0.572	0.614
Litter weight, kg	18.96	19.70	0.334	0.109	< 0.001
Post cross-foster measurements					
Litter size, *n*	13.42	13.72	0.186	0.242	0.077
Litter weight, kg	18.84	19.29	0.294	0.266	< 0.001
Weaning measurements^6^					
Sows weaned, *n*	105	106	---	---	---
Weaning age	19.39	18.50	0.096	< 0.001	0.523
Litter size, *n*	11.84	12.04	0.168	0.381	0.125
Survival after cross-foster, %	88.86	88.59	1.160	0.863	0.511
Litter weight, kg	65.90	64.86	1.139	0.507	< 0.001
21-day adjusted litter weight,^7^ kg	71.99	73.35	1.245	0.429	< 0.001
Piglet body weight,^8^ kg					
Born alive	1.40	1.43	0.021	0.281	0.017
12-hour	1.41	1.44	0.021	0.325	0.025
After cross-foster	1.41	1.44	0.021	0.375	0.031
Weaning^6^	5.59	5.40	0.076	0.071	< 0.001
21-day adjusted weaning	6.11	6.10	0.083	0.976	< 0.001
Litter treated for scours,^9^ %	45.55	12.24	3.957	< 0.001	< 0.001

^1^ Individual piglet (PIC 1050 × 280) data was collected on progeny of an additional 211 sows to determine if feeding a whole-cell inactivated yeast product (WCY; ADM Alliance Nutrition, Inc., Quincy, IL), to sows in gestation and lactation diets influences sow and pre-weaning pig performance in a commercial production system. Data were analyzed as a completely randomized design using the MIXED procedure in SAS 9.4 (SAS Institute, Inc., Cary, NC) with sow or piglet as the experimental unit. For sow, litter, and individual piglet performance, dietary treatment of the sow and parity (1 to 7) were fixed effects. No dietary treatment ×parity interaction was observed and therefore, the interaction was removed from the model.

^2^ Two maternal dietary treatments were fed. In gestation, post pregnancy check on d 35, and in lactation, sows were fed a control basal diet or the basal diet with 0.15% added WCY replacing corn.

^3^ Total born was calculated as born alive plus stillborn piglets and does not include mummies.

^4^ Data were square root-transformed to meet assumptions of normality; however, means and standard errors are presented as non-transformed values for ease of interpretation and p-values represent the square root-transformed data.

^5^ The 12-hour measurements were collected between 1 and 12 hours of birth from living piglets and before cross-fostering. The 12-hour survival rate does not include stillborn mortality.

^6^ Weaning weights were collected 1 day before actual weaning age.

^7^ Due to a significant difference in weaning age, weaning litter weights were adjusted for weaning age to a 21-day basis using adjustment factors from the Guidelines for Uniform Swine Improvement Programs 10.2.1 written by the National Swine Improvement Federation in December 1987.

^8^ Piglet body weights were calculated by dividing the litter weight by the number of pigs in the litter. Born alive is the litter weight at birth not including stillborn pigs. The 12-hour weight is the litter weight before cross-fostering, which would exclude any crushed or non-viable pigs at that time. Weaning is the litter weight at weaning. The 21-day adjusted weaning is the 21-day adjusted litter weight divided by the number of pigs at weaning.

^9^ Percent of litters treated for scours at least 1 time.

Pig body weights were calculated by dividing the whole litter weight by the number of pigs in the litter at each time point. Average born alive weight and weight at 12 h was not different due to treatment (*P* > 0.12; Table 2). The average weaning weight was 0.35 kg heavier for pigs from CON fed sows compared to WCY fed sows (*P* < 0.001). However, WCY litters were 0.93 d younger than CON litters (*P* < 0.001). Therefore, average weaning weights of litters were adjusted to 21 d of age using adjustment factors from the Guidelines for Uniform Swine Improvement Programs 10.2.1 (National Swine Improvement Federation, 1987). After this adjustment, the average 21-d adjusted individual pig weaning weight was still greater for CON litters compared to WCY litters by 0.18 kg (*P* < 0.012). The whole litter 21-d adjusted weaning weights were similar (*P* > 0.158), but numerically greater, for the WCY sows. Therefore, this increase in 21-d adjusted weaning weight of pigs from CON fed sows may be due to the smaller litter size throughout lactation of CON fed sows. The percent of litters treated for scours decreased from 38.3 to 14.2% when sows were fed WCY (*P* < 0.001).

A subset of ~100 litters per dietary treatment were weighed on an individual pig basis (Table 3).Within this subset, pigs were weaned 0.89 d earlier from WCY sows compared to CON (*P* < 0.001) and the percent of litters treated for scours decreased from 45.6 to 12.2% when sows were fed WCY, all similar values to the whole 300 sow data set described above (*P* < 0.001). There were trends for increased born alive litter weight (*P* < 0.063) but decreased individual pig weaning weight (*P* < 0.071) when sows were fed WCY compared to pigs and litters from CON fed pigs. However, when weaning weights were adjusted to 21 d, individual pig weaning weights were nearly identical between treatments (6.11 vs. 6.10 kg). This 100 sows/treatment subset had a similar, though non-significant, 0.40 piglet increase in total born (*P* < 0.320; Table 3) whereas the total dataset with about 300 sows/treatment had a 0.45 piglet increase in total born (*P* < 0.04; Table 2).

### Parity Effects

There were no parity by treatment interactions, therefore the parity main effects are being provided as additional information generated from the data in this experiment and had no effect on the dietary treatment results presented above. For the whole 600+ sow data set, there was an effect of parity (*P* < 0.049) for all performance parameters except for the number of mummies in a litter (*P* < 0.244) and weaning age tended to be affected by parity (*P* < 0.092; Table 4). Parity 1 sows had fewer total born pigs compared to all other parities (*P* < 0.008) and parity 6 sows had fewer total born pigs compared to parity 5 sows (*P* < 0.033). Parity 1 sows had fewer born alive pigs and a lighter born alive litter weight compared to parities 2, 3, 4, and 5 (*P* < 0.01). Parity 6 and 7 sows had fewer born alive pigs compared to parities 3 and 5 (*P* < 0.033). Parity 1 sows had a fewer number and lower percent stillborn piglets compared to all other parities (*P* < 0.045), except parity 2 (*P* > 0.05). Parity 7 sows had more stillborn pigs compared to parities 1 and 2 (*P* < 0.007). Parity 7 sows had a larger percent stillborn compared to parities 1, 2, and 3 (*P* < 0.039). Parity 5 sows had a lighter born alive litter weight compared to parities 2 and 3 (*P* < 0.003). Parity 1 and 6 sows had a lighter born alive litter weight compared to parities 2, 3, 4, and 5 (*P* < 0.028). Parity 7 sows had a lighter born alive litter weight compared to parities 2, 3, and 4 (*P* < 0.001).

Parity 1 sows had a higher litter percent survival at 12 h before cross-foster compared to parities 4, 5, and 6 (*P* < 0.028) and parity 2 sows had a greater litter percent survival than parity 6 (*P* < 0.045). Parity 1 sows had lighter litter weights at 12 h compared to parities 2, 3, 4, and 5 (*P* < 0.001). Parity 2 and 3 sows had heavier litter weights at 12 h compared to parities 5, 6, and 7 (*P* < 0.003). Parity 4 sows had heavier litter weights at 12 h compared to parities 6 and 7 (*P* < 0.003) and parity 5 had heavier litter weights at 12 h than parity 6 (*P* < 0.013). Parity 1 and 6 sows had fewer pigs in the litter at 12 h post-farrow compared to parities 2, 3, and 5 (*P* < 0.006). Parity 4 sows had fewer pigs in the litter at 12 h post-farrow compared to parity 3 sows (*P* < 0.029). Parity 2 and 3 sows had more pigs in the litter post cross-foster compared to parities 4, 6, and 7 (*P* < 0.040).

Parities 1 and 2 sows had a higher percent survival from cross-foster to weaning compared to parities 3 and 7 (*P* < 0.026). Parity 1 sows had a lighter litter weaning weight and 21-d adjusted litter weaning weight compared to all other parities (*P* < 0.001), except parity 7 (*P* < 0.284). Parity 2 sows had heavier litter weaning weights compared to parities 5, 6, and 7 (*P* < 0.002). Parities 3 and 4 had heavier litter weaning weights compared to parities 6 and 7 (*P* < 0.010), and parity 5 had heavier litter weaning weights than parity 7 (*P* < 0.003). Parities 2 and 4 sows had heavier 21-d adjusted litter weaning weights compared to parities 5, 6, and 7 (*P* < 0.034). Parity 3 sows had heavier 21-d adjusted litter weaning weights compared to parities 6 and 7 (*P* < 0.006) and parity 5 sows were greater than parity 7 (*P* < 0.006). Parity 2 sows weaned more pigs than parities 4, 5, 6, and 7 (*P* < 0.009). Parity 1 and 3 sows weaned more pigs than parity 6 and 7 sows (*P* < 0.007). Parity 4 and 5 sows weaned more pigs than parity 7 sows (*P* < 0.017).

Parity 1, 5, and 7 sows had a lighter average pig born alive weight compared to parities 2, 3, and 4 (*P* < 0.003). Parities 2 and 4 sows had heavier average pig born alive weights compared to parities 5, 6, and 7 (*P* < 0.010). Parity 1 sows had lighter average pig weaning weights and 21-d adjusted average pig weaning weights compared to all other parities (*P* < 0.001). Parity 4 sows had heavier average pig weaning weights compared to parities 3, 5, 6, and 7 (*P* < 0.025). Parity 4 sows had heavier 21-d adjusted average pig weaning weights compared to all other parities (*P* < 0.031). Parity 1 sows had a greater percent of litters treated for scours compared to all other parities (*P* < 0.001). Parity 2 sows had a greater percent of litters treated for scours compared to parity 6 sows (*P* < 0.030).

### Modeling Individual Pig Data

Individual birth weights were collected on 2,960 pigs of which 2,507 survived to weaning (84.7%). Individual birth weight means were adjusted for parity group (1, 2, and 3+) as a fixed effect and total born was used as a covariate. Although in Tables 3 and 4 there were no difference in birth weights of live born pigs between treatments (*P* > 0.12), when adjusting for parity group and total born, pigs from sows fed WCY were heavier compared to CON (1.43 kg vs. 1.39 kg; *P* < 0.001). For every 1 additional pig born, birth weight per pig decreased 0.037 kg (*P* < 0.001). Pigs born to parity 1 sows were 0.092 kg lighter compared to parity 2 and 3+ sows (*P* < 0.001).

**Figure 1:**
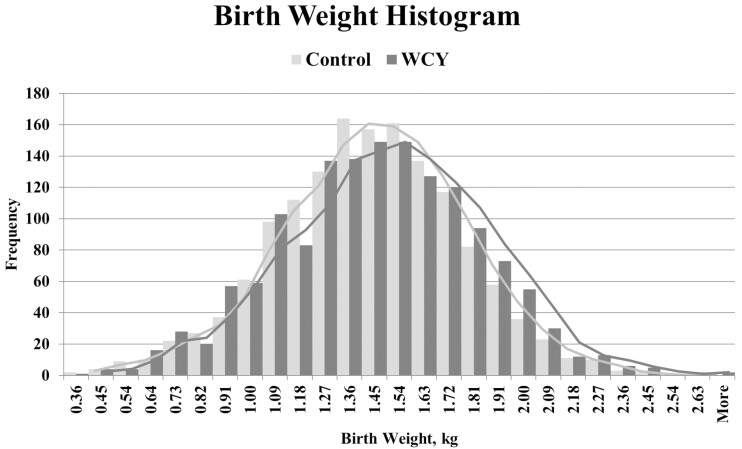
Birth Weight Histogram. Sows were fed a control basal diet or the basal diet with 0.15% added whole cell yeast (WCY) in gestation starting on d 35 post pregnancy check through lactation. Individual piglet birth weights (*n* = 2960) were divided into ranges separated at 0.09 kg increments. The distribution of birth weights by birth weight range was not different (chi-squared; *P* < 0.246) between sow dietary treatments.

**Figure 2:**
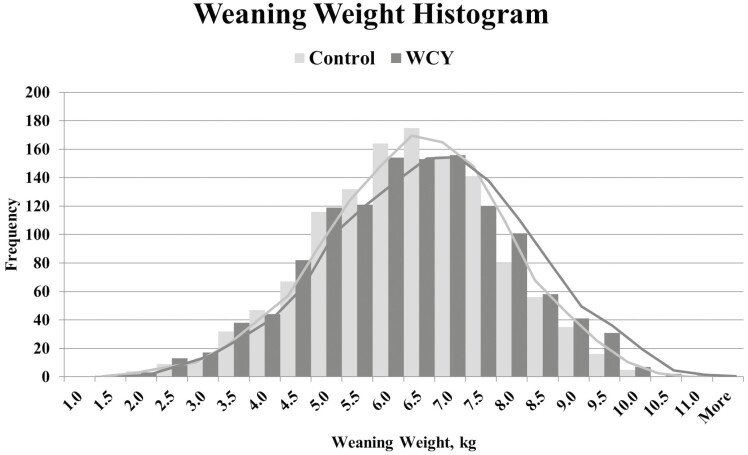
Weaning Weight Histogram. Sows were fed a control diet or the control diet with 0.15% added whole cell yeast (WCY) in gestation starting on d 35 post pregnancy check through lactation. Individual piglet weaning weights (*n* = 2507) were divided into ranges separated at 0.50 kg increments. The distribution of weaning weights by weaning weight range was not different (chi-squared; *P* < 0.355) between sow dietary treatments.

To visualize the distribution of birth weights, individual pig birth weights were divided into ranges in 0.09 kg increments. The distribution of birth weights by weight range was not different (*P* < 0.2461; Figure 1) between sow dietary treatments. Individual pig weaning weights were divided into ranges separated at 0.50 kg increments. The distribution of weaning weights by weight range was not different (*P* < 0.3551; Figure 2) between sow dietary treatments.

**Figure 3:**
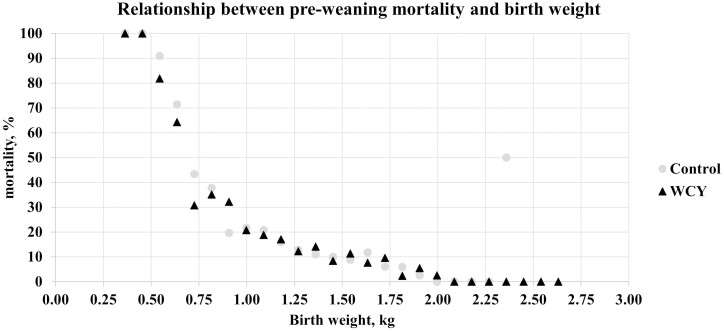
Relationship between pre-weaning mortality and birth weight. Sows were fed a control diet or the control diet with 0.15% added whole cell yeast (WCY) in gestation starting on d 35 post pregnancy check through lactation. Individual piglet birth weights (*n* = 2960) were divided into weight ranges separated at 0.09 kg increments. Within each birth weight range, the proportion of piglets that did not survive was calculated and presented as percent mortality. The 2.36 kg birth weight range for the control treatment was comprised of only 2 pigs in which one piglet did not survive to weaning.

To visualize the relationship between birth weight and mortality, raw data were used to group birth weights into 0.09 kg weight ranges and the proportion of pigs that died within each weight range was plotted (Figure 3). Although not statistically analyzed, the percent mortality decreased as birth weight increased. Pigs weighing less than 0.61 kg at birth had less than a 40% survival rate.

There was no treatment effect (*P* > 0.05) on standardized 21 d litter weaning weights, however there were effects due to all other factors included in the model detailed in the statistics section of this paper (*P* < 0.001). Pigs born to parity 1 sows weighed 0.837 kg lighter at weaning compared to parities 2 and 3+ (*P* < 0.001). For every 0.1 kg increase in birth weight, pigs weighed 0.441 kg heavier at weaning (*P* < 0.001). For every 1 pig increase in litter size post cross-foster, pigs weighed 0.12 kg lighter at weaning (*P* < 0.001). Last, if pigs were cross-fostered to another litter, they were 0.58 kg lighter at weaning (*P* < 0.001). This decrease in weaning weight due to cross-fostering held true for pigs cross-fostered up to 2.03 kg of birth weight (Table 5).

**Table 5.** Effects of cross-fostering on weaning weight^1^.

	Average Wean Weight (*n*)		Difference, kg
	Cross-fostered	Not Cross-fostered	
Birth weight range, kg			
0.61 – 1.12	4.01 (9)	4.65 (389)	0.64
1.13 – 1.58	5.15 (32)	5.88 (1189)	0.73
1.59 – 2.03	5.97 (27)	6.93 (753)	0.96

^1^ Modeling adjusted 21-day weaning weights of individual piglets that survived to weaning using the GLM procedure of SAS 9.4. Dietary treatment, parity group, birth weight within treatment, post cross-foster litter size, and if the piglet was cross-fostered or not were all included. Average weaning weights within each birth weight range was calculated and number of pigs in each category noted.

**Figure 4:**
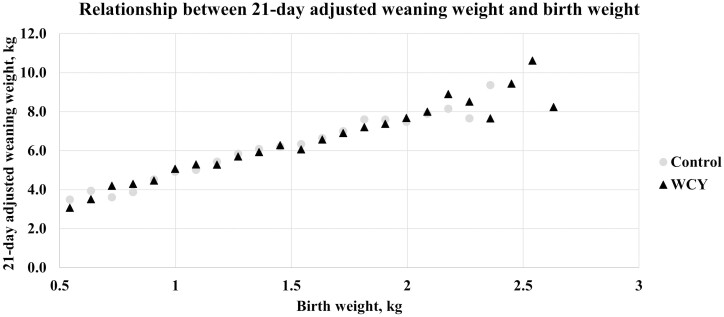
Relationship between 21-day adjusted weaning weight and birth weight. Sows were fed a control diet or the control diet with 0.15% added whole cell yeast (WCY) in gestation starting on d 35 post pregnancy check through lactation. Individual piglet birth weights (*n* = 2507) were divided into weight ranges separated at 0.09 kg increments. Within each birth weight range, the average 21-d adjusted weaning weight was calculated. Due to a significant difference in weaning age between treatments, individual weaning weights were adjusted for weaning age to a 21-day basis using adjustment factors from the Guidelines for Uniform Swine Improvement Programs 10.2.1 written by the National Swine Improvement Federation in December 1987. The generalized regression reveals for every 0.1 kg increase in birth weight, piglet weaning weight increased by 0.441 kg (*P* < 0.001).

**Figure 5:**
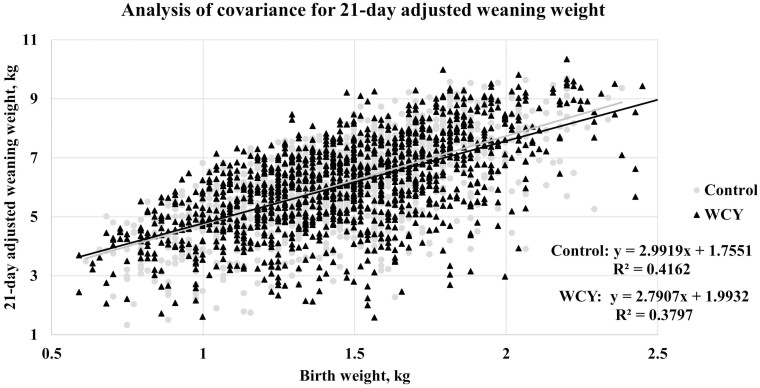
Analysis of covariance for 21-day adjusted weaning weight. Sows were fed a control diet or the control diet with 0.15% added whole cell yeast (WCY) in gestation starting on d 35 post pregnancy check through lactation. Due to a significant difference in weaning age between treatments, individual weaning weights (*n* = 2507) were adjusted for weaning age to a 21-day basis using adjustment factors from the Guidelines for Uniform Swine Improvement Programs 10.2.1 written by the National Swine Improvement Federation in December 1987. In a general linear model, birth weight and sow dietary treatment explained 39.6% of the variation in 21-day adjusted weaning weight (average R^2^ = 0.396). Birth weight was significant in the model (*P* < 0.001), however sow dietary treatment (*P* < 0.26) and the interaction of birth weight and treatment (*P* < 0.16) were not significant.

To visualize the relationship between 21-d adjusted weaning weight and birth weight, birth weights were grouped into 0.09 kg weight ranges and the average adjusted weaning weight within each birth weight range was plotted for each dietary treatment (Figure 4). When modeling 21-d adjusted weaning weight using a general linear model with birth weight and dietary treatments as independent variables, the overall model was significant (*P* < 0.001) where weaning weight increased as birth weight increased for both dietary treatments. Within the model, birth weight was significant (*P* < 0.001), however treatment (*P* < 0.26) and the interaction of birth weight and treatment (*P* < 0.16) were not significant. Birth weight and dietary treatment explained 39.6% of the variation in 21-d adjusted weaning weight (average *R*^2^ = 0.396; Figure 5).

## DISCUSSION

Research investigating *Pichia guilliermondii* products fed to sows is fairly limited when compared to the common yeast, *Saccharomyces cerevisiae*, derived products. Veum et al. (1995) fed sows *Saccharomyces cerevisiae* yeast culture from d 60 of gestation through d 21 of lactation at 0, 0.5, 1.0, or 2.0% of complete feed and observed no differences in sow reproductive performance. When sows were fed a *Saccharomyces cerevisiae* fermentation product 5 d before breeding through lactation (12 g/d in gestation and 15 g/d in lactation), Shen et al. (2011) observed no differences in reproductive performance of the sow. However, sows fed the *Saccharomyces cerevisiae* fermentation product tended to have heavier litter weights at weaning and increased litter body weight gains compared to litters from control fed sows, which could be due to yeast fed sows weaning numerically more pigs (10.3 vs. 9.2; Shen et al., 2011).

Bass et al. (2019) fed sows an inactivated, whole yeast cell *Pichia guilliermondii* product at 0, 0.1, or 0.2% of complete feed within 24 h of breeding through the 21-d lactation period. Sows were housed in individual gestation stalls and individual farrowing crates. Inclusion of *Pichia guilliermondii* in the sow diet linearly increased the number of pigs born alive per litter as the dietary inclusion increased (12.49, 13.33, and 13.43 for 0, 0.1, or 0.2%, respectively; Bass et al., 2019).

Data from the current study supports findings of Bass et al. (2019). The addition of the inactivated, whole cell yeast *Pichia guilliermondii* product at 0.15% of the sow diet in this study resulted in a greater number of piglets born alive. The experiment by Bass et al. (2019) was comprised of about 30 sows per treatment in a university research setting using gestation stalls whereas the current study collected data on about 300 sows per treatment in a commercial swine production system where sows were housed in gestation pens. Interestingly, the increase in the number of pigs born alive was consistent across the two very different studies when an inactivated, whole cell yeast *Pichia guilliermondii* product was fed.

Sows in the current study were housed in gestation stalls and fed diets without yeast supplementation for 35 d before being confirmed pregnant. Once confirmed pregnant, they were moved into gestation pens with electronic sow feeders and fed the control diet or the diet supplemented with 0.15% *Pichia guilliermondii* whole cell yeast (WCY). Sows fed the WCY had an increased number of pigs total born and born alive compared to CON fed sows. Because sows were fed the same diets until d 35 of gestation, it is logical to assume the two groups of sows had similar ovulation rates and conceptus implantation success because both of these events occur before d 35 of gestation (Ziecik et al., 2011).

According to Ford et al. (2002), 30–50% of fertilized ova do not make it to term in U.S. pig breeds and 75% of these losses occur before d 30 of gestation. There are two additional periods of significant conceptus loss in sows which occur from d 30-40 and d 90-114 of gestation when uterine capacity, including space and nutrients, becomes a critical limiting factor due to competition among litter mates (Ford et al., 2002). These two time periods are defined by abrupt increases in surface area between the placenta and uterine luminal surface where microscopic interdigitations develop known as primary and secondary rugae (Bjørkman and Dantzer, 1987). The exact mechanism of how the mannan-oligosaccharide and β-glucan cell wall components of the WCY may impact conceptus survival due to uterine capacity at these two time periods is unclear. It is also possible that this dietary yeast product contains other undiscovered active *Pichia guilliermondii* culture components that may be residual from the citric acid fermentation process.

Bass et al. (2019) observed an increase of 0.94 pigs born alive when sows were fed 0.2% *Pichia guilliermondii* starting at breeding compared to litters from sows not fed any yeast products. The current study fed 0.15% *Pichia guilliermondii* WCY to sows starting on d 35 of gestation and an increase of 0.42 pigs born alive was observed versus CON fed sows. The response observed by Bass et al. (2019) was two times the response observed in the current study which could be due to the larger dose of WCY, longer duration of feeding the *Pichia guilliermondii* WCY, and feeding it during a critical time frame of embryo loss before d 30 of gestation. Additionally, the litter size of control fed sows in the current study was 1.36 pigs larger than Bass et al. (2019) which may have decreased the potential for born alive litter size improvement in the current study.

In recent decades, genetic selection, advanced nutrition, and improved reproductive technologies have successfully increased litter sizes to 12-15 piglets born alive. This increase in litter size has resulted in a greater variation of within litter birth weights and a greater proportion of light weight pigs at birth (less than 1 kg BW; Quesnel et al., 2008). In the current study, sows fed WCY had a larger litter size than controls, but the expected greater proportion of light weight pigs was not observed. Previously, Bass et al. (2019) fed an inactivated, whole yeast cell *Pichia guilliermondii* product to sows and reported a smaller proportion of light weight pigs from those sows compared to sows not supplemented with yeast products.

Lighter birth weight pigs have a greater chance of pre-weaning mortality. These small pigs lose body heat more rapidly, have smaller glycogen pools, and smaller stomachs which limits colostrum consumption especially when trying to compete within a large litter (Theil et al., 2012). In this study, pigs weighing less than 0.61 kg at birth had less than a 40% survival rate. In agreement, Feldpausch et al. (2019) evaluated data including 4,068 pigs from 394 litters on four commercial farms and reported that 15.2% of pigs weighed less than or equal to 1.11 kg at birth and these pigs had a 34.4% mortality rate representing 43% of all pre-weaning mortalities.

As previously stated, it is logical to assume the two groups of sows in the current experiment had similar ovulation rates and conceptus implantation success because both of these events occur before d 35 of gestation (Ziecik et al., 2011). Therefore, one might expect to observe an increased number of mummies in litters of the CON sows because they had fewer total born pigs. However, in this study, there was no difference in the number of mummies per litter due to dietary treatment. According to Flowers (2019), calcification of fetal bones begins at about d 38-45 of gestation and fetal losses after this point result in formation of a mummified fetus due to the failure of the uterus to reabsorb bone. It is possible that CON fed sows had more conceptus loss from d 35-45 in which those losses did not result in formation of mummies. It is also possible that mummies formed a short time period after d 45 may not have been large enough to be detected in a commercial production system.

In the current study, sows fed *Pichia guilliermondii* gave birth to more pigs resulting in a heavier born alive litter weight compared to CON fed sows. When Kim et al. (2008) supplemented sow diets with a *Saccharomyces cerevisiae* yeast culture starting on d 35 of gestation (12 g/d) until d 21 of lactation (15 g/d) there was no difference in litter size at birth or litter birth weight. However, authors did observe an increased litter weaning weight due to supplemental *Saccharomyces cerevisiae* yeast culture. Although milk samples were not collected, Kim et al. (2008) speculated the increased litter wean weight could be due to increased production of milk and/or of higher quality milk when sows were fed the yeast culture product. In contrast, no differences in 21-d adjusted litter weaning weights due to dietary treatment were observed in this experiment. In agreement with the current study, Bass et al. (2019) observed no differences in total litter or average individual weaning weights with increasing WCY from 0 to 0.2%.

In this experiment, pre-cross-fostering survival was not different between dietary treatments and cross-fostering within treatments occurred after d 0 care and weights to equalize litter size. Since WCY fed sows had larger litter sizes at birth and there were no differences in survival before cross-fostering, CON fed sows had 0.68 fewer pigs per litter to nurse during lactation. Sows fed the WCY diet weaned 0.54 more pigs per litter compared to CON fed sows. The smaller number of pigs nursing CON sows were potentially consuming more milk nutrients per pig which could be one reason why individual CON pigs were heavier at weaning.

Furthermore, 38% of CON litters (114 of 300 total litters) were treated for *E. coli* scours whereas only 14% of WCY litters (43 of 306 total litters) were treated. Although diarrhea is known to have negative impacts on growth performance up to the first week of life (Moeser et al., 2017), the antibiotic treatment (at least one dose of spectinomycin) could have helped eliminate other subclinical challenges in the farrowing house allowing pigs from CON fed sows to grow better thereafter to weaning. There was no difference in pre-weaning mortality between dietary treatments. However, if pigs did not receive spectinomycin on this farm, a decreased survival to weaning likely would have been observed for CON litters compared to WCY litters. Although not specific to *E. coli* scours, a Swine 2012 USDA report found that 10% of all preweaning mortality is from scours (USDA, 2015).

Differences in reproductive and litter performance were detected due to dietary treatment and sow parity. Parity 1 sows had the fewest total born pigs per litter (13.7) compared to all other parities (average of 15.2) and fewer pigs born alive per litter (13.2) than parities 2 through 5 (average of 14.5). In agreement sows in parities 2 through 5 had larger litter sizes born alive (average of 11.3) than parity 1 and parity 8 sows (average of 10.7; [Bibr CIT0015][Bibr CIT0015]). Although litter sizes have increased with genetic selection, parity 3 to 5 sows appear to still be the most productive animals ([Bibr CIT0026][Bibr CIT0026]).

Litter sizes may be smaller due to parity 1 sows having a smaller uterine capacity which limits the number of developing fetuses (Sell-Kubiak et al., 2019). In geriatric sows (parity 6 and older), lower reproductive performance can be attributed to decreased ovulation and fertilization rates and higher embryonic mortality due to slower responses to fetal demands for uterine space ([Bibr CIT0016][Bibr CIT0016]). Lower reproductive performance of geriatric sows could also be due to the inadequate amounts of micronutrients provided in the diet on a per kg body weight basis. More micronutrients provided to the heavier sows may help to support tissue metabolism and combat the age-related decline in litter size (Boyd et al., 2008). Additionally, observed in the present study, parity 7 sows had more stillborn pigs per litter (1.4) compared to parities 1 and 2 (0.55 and 0.70, respectively).

Kim et al. (2008) reported that primiparous sows produced litters with lighter birth weights compared to multiparous sows. Litters from primiparous sows were also heavier at weaning than multiparous sows, but the heavier weaning weight could be due to about a half of a pig increase in litter size at weaning for litters from primiparous sows (Kim et al., 2008). In the current study when all 606 sows were included, there was a parity main effect for born alive litter weight and 21-d adjusted litter weaning weight. In agreement with Kim et al. (2008), parity 1 sows had a lighter born alive litter weight compared to parities 2, 3, 4, and 5, however there was no difference in born alive litter weight between parities 1, 6, and 7 in this experiment. In contrast to Kim et al. (2008), parity 1 sows in this experiment had a lighter 21-d adjusted litter weaning weight compared to all other parities, except parity 7. Interestingly, litter size at weaning was greater for parity 1 sows compared to parities 6 and 7, therefore parity 1 sows simply weaned smaller pigs than all other parities.

The observation that parity 1 sows have smaller pigs at birth and at weaning is common (Craig et al., 2017). After conception, gilts must continue to grow and prioritize nutrients toward their own growth and development (e.g. mammary growth) as well as fetal growth (Theil et al., 2012). Therefore, it’s logical that multiparous sows that prioritize more nutrients toward fetal growth would have larger pigs at birth and that larger framed, later parity sows would have the capacity to carry heavier litters. Considering weaning weight, a parity 1 sow compared to a multiparous sow will be physically smaller and therefore will have less stomach capacity for feed intake in lactation to support milk production. Milk yield of multiparous sows is typically greater than milk yield of primiparous sows which results in multiparous sows raising heavier pigs to weaning (Quesnel et al., 2015). Additionally, multiparous sows have more and better developed mammary glands that produce more milk compared to primiparous sows (Farmer, 2018).

There was also an effect of parity on the percentage of litters treated for scours between d 1 and 3 of life. Parity 1 litters were treated more often for scours than any other parity which may be due to parity 1 sows providing less passive immunity to their litters. Quesnel (2011) measured colostrum concentrations of immunoglobulin G (IgG) at 24 h after birth and found that IgG concentrations were greater in older sows than primiparous sows.

In conclusion, the addition of WCY to gestation diets after d 35 and lactation diets increased total number born by about half a pig per litter which increased the born alive litter weight compared to CON fed sows. Litter size at weaning remained increased by about half a pig per litter when sows were fed WCY compared to CON. However, individual pigs born to WCY fed sows had lighter weaning weights compared to pigs from CON fed sows even after adjusting for lactation length, which may be due to WCY sows nursing larger litters. The percent of litters treated for scours was decreased due to feeding WCY, adding to the economic, labor savings, and piglet welfare potential benefits of feeding WCY.

## Notes

Appreciation is expressed to ADM Animal Nutrition (Quincy, IL) for financial support of this study which also includes the CitriStim product used and feed analyses.
